# Reliability of Synaptic Transmission at the Synapses of Held *In Vivo* under Acoustic Stimulation

**DOI:** 10.1371/journal.pone.0007014

**Published:** 2009-10-02

**Authors:** Bernhard Englitz, Sandra Tolnai, Marei Typlt, Jürgen Jost, Rudolf Rübsamen

**Affiliations:** 1 Max Planck Institute for Mathematics in the Sciences, Leipzig, Germany; 2 Faculty of Biosciences, Pharmacy and Psychology, University of Leipzig, Leipzig, Germany; University of Washington, United States of America

## Abstract

**Background:**

The giant synapses of Held play an important role in high-fidelity auditory processing and provide a model system for synaptic transmission at central synapses. Whether transmission of action potentials can fail at these synapses has been investigated in recent studies. At the endbulbs of Held in the anteroventral cochlear nucleus (AVCN) a consistent picture emerged, whereas at the calyx of Held in the medial nucleus of the trapezoid body (MNTB) results on the reliability of transmission remain inconsistent. *In vivo* this discrepancy could be due to the difficulty in identifying failures of transmission.

**Methods/Findings:**

We introduce a novel method for detecting unreliable transmission *in vivo*. Based on the temporal relationship between a cells' waveform and other potentials in the recordings, a statistical test is developed that provides a balanced decision between the presence and the absence of failures. Its performance is quantified using simulated voltage recordings and found to exhibit a high level of accuracy. The method was applied to extracellular recordings from the synapses of Held *in vivo*. At the calyces of Held failures of transmission were found only rarely. By contrast, at the endbulbs of Held in the AVCN failures were found under spontaneous, excited, and suppressed conditions. In accordance with previous studies, failures occurred most abundantly in the suppressed condition, suggesting a role for inhibition.

**Conclusions/Significance:**

Under the investigated activity conditions/anesthesia, transmission seems to remain largely unimpeded in the MNTB, whereas in the AVCN the occurrence of failures is related to inhibition and could be the basis/result of computational mechanisms for temporal processing. More generally, our approach provides a formal tool for studying the reliability of transmission with high statistical accuracy under typical *in vivo* recording conditions.

## Introduction

Transmission at neuronal synapses is central to neuronal processing and its investigation has a long history. The small size of most central synapses renders detailed measurements difficult using current electrophysiological techniques. Two giant synapses in the auditory brainstem, the endbulbs of Held and the calyces of Held, allow better access due to their size. They serve as important model systems for synaptic transmission at central synapses and have been extensively studied in *in vitro* preparations (for review see [1]). The endbulbs of Held convey information from the auditory nerve to spherical bushy cells in the anteroventral cochlear nucleus (AVCN, [2]–[4]). The calyces of Held are formed by axons of cochlear nucleus globular bushy cells onto principal cells in the medial nucleus of the trapezoid body (MNTB, [2], [5]). The size of the presynaptic terminals allows simultaneous recording of the activity of the presynapses *in vitro* (chick AVCN homologue: [6], MNTB: [7]–[10]) and *in vivo* (AVCN: [11], [12]; MNTB: [13], [14]).

In both nuclei, the reliability of signal transmission has been investigated under different conditions, where failures in transmission were indicated by the occurrence of presynaptic potentials not followed by postsynaptic action potentials (APs). In the AVCN, *in vivo* and *in vitro* recordings indicate failures of transmission at the endbulbs of Held. *In vivo* auditory stimulation and pharmacological manipulation provided evidence for failures, suggesting the involvement of inhibitory inputs [11], [12]. *In vitro*, failures occurred during high frequency electrical stimulation of the excitatory afferents [15]. In contrast, studies in the MNTB yielded conflicting results. *In vitro* the signal transmission at the calyx is usually described as fast, secure, and reliable [16]–[18], but two recent studies [19], [20] indicated low firing rates and short duration stimulations in typical *in vitro* studies as a possible reason for the lack of failures. *In vivo* signal transmission failed at high electrical stimulation rates [13] and when auditory stimuli are presented that reduce the firing rate of MNTB neurons [14], but another recent study [21] suggested that these failures could be due to shortcomings in the analysis in [14].

The shortcomings pertain to the possibility of mistaking signals from other cells as incidences of failed transmission. This possibility arises due to the summation of signals in the extracellular fields and has been known to experimenters for a long time. It also arises in the task of distinguishing spikes emitted from different sources (spike-sorting) where single and multiunit activity need to be distinguished. The refractory period of neuronal spiking is a basic criterion to distinguish these alternatives. To detect incidences of failed transmission, a similar approach can be used. McLaughlin et al. [21] spike-sorted both the action potentials and failure candidates and compared their temporal relationship. While this analysis provides qualitatively similar results as presented here, it does not yield a statistical test. The presence of noise, however, necessitates such a test in order to avoid the time-consuming and possibly biased task of visually classifying recordings or interspike interval histograms as corresponding to failures of transmission or not.

The presently developed test also constructs a specific interspike interval histogram, but statistically compares the distribution within one refractory period to the distribution at greater distances. In this way it becomes robust to noise and a significance level of the decision can be computed. The properties of the test are first investigated and adjusted using simulated voltage recordings. It is then applied to a (comparably) wide range of recordings from the endbulbs and the calyces of Held. In summary, we suggest that under the studied activity conditions (spontaneous, excited, acoustically suppressed activity) and employed anesthesia (ketamine/xylazine) failures of transmission occur commonly in the AVCN but only rarely in the MNTB. The present results have partly been presented at the ARO midwinter meeting 2008 (Abstract #845).

## Materials and Methods

### Physiology

#### Ethics Statement

All animals were handled in strict accordance with good animal practice as defined by the relevant regional animal welfare body, and all animal work was approved by the appropriate committee (Landesdirektion Leipzig, TVV50/06).

#### Preparation

Forty adult pigmented Mongolian gerbils (Meriones unguiculatus, aged 2–4 months, weighing 45–70 g) were used in this study. They were anesthetized with a xylazine-hydrochloride/ketamine-hydrochloride mixture (xylazine (Rompun, Bayer Pharmaceuticals) 0.007 mg/g body weight injected intraperitoneally; ketamine (Ketavet, The Upjohn Company) 0.18 mg/g body weight initial dose by intraperitoneal injection). Subcutaneous, hourly injections of one third of the initial dose assured a constant level of anesthesia. Animals were placed in a sound-attenuated booth (Type 400, Industrial Acoustic Company) on a vibration-isolated table and fixed in a stereotaxic device using a metal bolt glued to the bone on bregma of the skull. The nuclei were approached dorsally with the animal tilted at 27–34 degrees (AVCN) and 4–10 degrees (MNTB) to the midsagittal plane. Further details of the preparation are given in [22].

#### *In vivo* localization of target nuclei

Stereotaxic coordinates of the AVCN (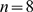) or the MNTB (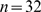) were determined by online analysis of acoustically evoked multiunit activity using low impedance micropipettes (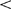
5 M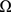). Locating the AVCN was facilitated by its clear tonotopic (spatial location of neurons varies systematically with preferred stimulus frequency) organization and the fact that units are only driven by stimuli presented to the ear on the same side. Differentiation of the MNTB from other nuclei within the superior olivary complex was facilitated by the fact that MNTB units increase their firing rate exclusively in response to stimuli presented to the ear on the opposite side. In 18 animals recording sites were additionally verified histologically using HRP or Flouro-Gold following the same procedures as in [22].

#### Single-unit recording

Extracellular voltage recordings were performed with high-impedance glass micropipettes (8–30 M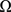, GB150TF-10, Science Products) filled with 3 M KCl. These recordings are similar to loose patch recordings (on-cell microelectrode recordings without a resistive seal in the G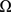 range and no breach of the cell membrane) due to the proximity of the micropipette tip to the cellular surface. In contrast to local field potential recordings, where the recording electrode is further from the cell membrane, the waveform properties correspond here to a bandpass filtered version of the transmembrane voltage (own unpublished observation). The voltage signal was preamplified (Neuroprobe 1600, A-M Systems, Carlsborg), band pass filtered (0.3–7 kHz), and further amplified (PC1, TDT, Alachua) to match the input voltage range of the A/D converter (RP2.1, TDT). Voltage traces were digitized at a sampling rate of 97.7 kHz and stored for subsequent analysis.

**Figure 1:**
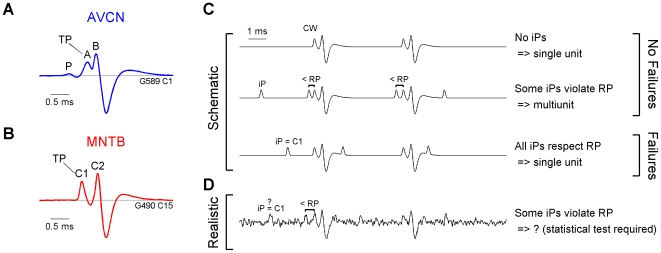
Complex Waveforms (CW) in AVCN and MNTB and overview of the general task. (A) In the AVCN the CW usually has three components, P, A, and B [11]. Aside from this CW, also the combination of only P and A occurs, constituting candidates of failed transmission. Since A is usually larger than P, the height of A was used for triggering the P-A combinations. Hence, in the AVCN A is the trigger potential (TP). (B) In the MNTB the CW has two components, C1 and C2 [13]. Here, the height of the presynaptic C1 serves to detect failure candidates, i.e. constitutes the TP here. (C) To check the reliability of transmission, three cases need to be distinguished (here illustrated without noise for a mean CW from an MNTB unit): (top) If the CW has a strong C1 component, but no potentials of a similar height (iPs) occur in the remaining trace, then the recording is from a single unit and there is no indication for failures. (middle) If iPs occur, but some of them are located too close (less than the refractory period, RP) to their counterpart (C1) in the CW, then the recording is from multiple units and the iPs do not stem from failures. (bottom) If iPs occur and they all respect the RP, then they likely correspond to failures (assuming other correlation factors, e.g. phase locking, are ruled out). (D) Under realistic recording conditions, iPs could be due to failures (only C1), other cells, or just noise fluctuations. Classical spike-sorting cannot reliably distinguish between these cases. If an iP is detected close to a C1, failures cannot necessarily be excluded since this could have been a noise fluctuation. To decide whether these violations are due to noise, a statistical test is required that compares the distribution of iPs at different distances from C1. In the AVCN, a corresponding argument would replace C1 with the P-A waveform.

Single units of the desired kind were identified by the characteristic shape of their waveform. Due to the extraordinary size of the synapses of Held, spike transmission at these junctions produces complex waveforms (CWs) which distinguish them from other cell types and fibers in these nuclei. Only units exhibiting such CWs were included in the analysis. In the AVCN, Pfeiffer [11] was the first to report the typical occurrence of a CW in extracellular recordings (Figure 1A). He separated it into three components termed P, A, and B. P and A are positive potentials with a relatively constant delay of 0.5 ms between them. B's shape concords with a typical extracellular action potential waveform and follows A with a considerably smaller and more variable delay (0–0.2 ms). Based on the temporal relationships, Pfeiffer concluded that P is of presynaptic origin, whereas A and B are postsynaptic events. In the MNTB, Guinan et al. [23] found similar CWs in extracellular recordings, consisting of two components separated by 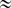
0.5 ms (Figure 1B). A number of studies [13], [24], [25] showed convincingly that the first component (C1) can be attributed to the presynaptic AP and the second component (C2) to a postsynaptic component, presumably the AP.

The interpretation of the present results will depend on the origin of the components of the CW. Especially in the AVCN this origin is not fully understood. Possible origins for the individual components include the presynaptic AP, the postsynaptic EPSP, the postsynaptic axon hillock AP and the postsynaptic, retrograde AP [11]. In the following we have attempted to report the results without favoring either interpretation.

The signal-to-noise ratio (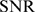) of potentials was quantified via the peak signal-to-noise ratio, defined as 
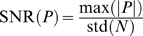
, where 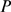 denotes a potential and 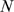 the baseline noise.

### Stimulus presentation

Reliability of transmission was investigated under three response conditions: (1) spontaneous, (2) excited, and (3) suppressed activity. For the latter two, specific acoustic stimuli were presented to the animal which achieved the desired activity level for a given cell (see below). During condition (1) no acoustic stimulus was presented. Spontaneous activity was recorded for 30 s, in units with low spontaneous activity for up to 150 s. For condition (2), the combinations of level and frequency leading to significant increases in firing rate were selected from the (stimulus frequency - stimulus intensity) tuning recordings (next paragraph). The length of these recordings varied with the response area of each unit, amounting to 40 s on average. In condition (3), either a single or a two-tone paradigm (two tonal stimuli presented together) was chosen depending on the spontaneous rate of the unit (next paragraph).

The excitatory response area of a neuron was measured by monaural presentation of short pure tones (100 ms, 200 ms stimulus period) in pre-defined intensity and frequency arrays. Combinations of 20 frequencies (logarithmic spacing) and 10 intensity levels (linear spacing in decibel) were presented in a randomized order. In spontaneously active units this single tone paradigm sufficed to determine significant reductions of (spontaneous) firing rate (Wilcoxon signed ranks test). In spontaneously inactive units a two-tone paradigm was used to detect frequency/intensity combinations evoking reductions in response rate relative to the response rate of a single excitatory tone (first, excitatory pure tone: at the characteristic frequency (CF, the tone frequency a unit is most sensitive at) with a duration of 100 ms; second pure tone: varying frequency/intensity combinations with a duration of 40 ms starting at a 30 ms delay with respect to the first tone within an overall stimulus period of 200 ms). All tones were modified multiplicatively at their start and end with cosine shaped rise/fall ramps (5 ms each). After the rate-reducing frequency/intensity combinations were located, they were resampled with longer stimuli to increase the spike count for this condition (CF tone: 500 ms duration, additional pure tone: 450 ms duration, 50 ms delayed to the CF tone, 700 ms stimulus period, providing a recording time of up to 75 s).

Stimulus waveforms were generated at a sampling rate of 97.7 kHz on a standard PC using custom written Matlab 7 (The Mathworks, Natick) software. Stimuli were then transferred to a real-time processor (RP2.1, Tucker Davis Technologies), D/A converted and further sent to two custom made earphones (acoustic transducer: DT 770 pro, Beyerdynamic). These were fitted with plastic tubes (35 mm length, 5 mm diameter) which were inserted into the outer ear canal at a distance of 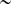
4 mm to the tympanic membrane. Acoustic calibration was performed by convolving the stimulus with the earphone's inverse impulse response prior to stimulus presentation. The calibration was verified to lie within 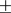
5 dB of the target amplitude in the range of 0.5 to 48 kHz before the experiment.

### Data analysis

The goal of the following analysis is to decide whether an MNTB/AVCN recording contains failures of spike transmission (illustrated in Figure 1C & D). The following decision sequence needs to be checked:

1. Do voltage potentials exist, which qualify as remnants of a transmission event, e.g. isolated presynaptic potentials?
  - If not, we can conclude that transmission is safe (based on the underlying assumptions).
  - If such potentials are present address next question:
2. Do they originate from a source which is connected to the CW's source?
  This is tested by determining whether the potentials obey the refractory period with respect to the CWs.
  - If this is the case, it is likely that these potentials originate from the presynaptic ending (if certain shape criteria match).
  - If the refractory period is not obeyed, the potentials have to originate from a different source, e.g. neighboring cells.

Recordings are divided into two classes, based on where they fall along these conditions: Failures of transmission are unlikely if either no candidate potentials exist or if the candidates stem from a different source. Failures of transmission are likely if the potentials stem from the same source. We decided to use a more cautious terminology. Rather than naming these classes *No Failures* and *Failures*, we chose the terminology *no dependent potentials* and *dependent potentials*, which will be abbreviated *No Dep* and *Dep*, respectively. This terminology reflects the basic fact that an analysis based on the waveforms can only determine whether the CWs and the candidate potentials are statistically independent or dependent. Their origin needs to be addressed later, based on additional analytical and biological considerations, which we postpone to the discussion.

**Figure 2:**
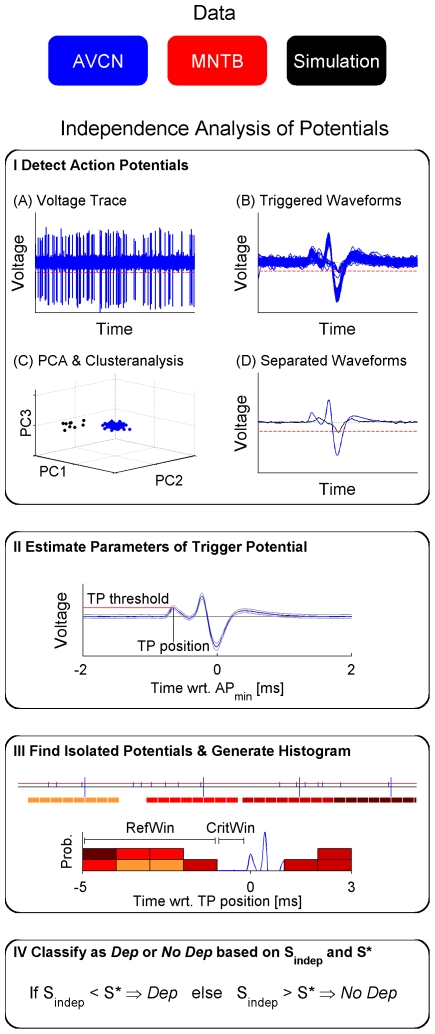
Schematic of the Independence Analysis of Potentials (IAP). We subjected three data sets to the IAP: voltage recordings from AVCN, MNTB, and simulations. I. First, candidate waveforms were collected by triggering at a visually chosen threshold (A, red) and then (B) aligned at their minimum. (C) Next, these waveforms were spike sorted using principal component and cluster analysis. (D) The cluster containing the complex waveforms (CWs, blue) was selected, thus excluding waveforms of different shape (black). II. The height and relative position of the trigger potential (TP  =  prepotential in MNTB;  =  A-component in AVCN) were detected in the average CW. (blue: mean; gray: ±1 SD) III. Isolated potentials (iPs, small vertical bars in upper graph) were triggered at the TPs height (red horizontal line) and collected into a histogram relative to the TP of the CW. Each box color corresponds to an iP preceding a given spike. Different colors indicate which CW they correspond to (compare to upper graph, box size does not indicate actual bin-size). IV. Whether the iPs derive from the CW's source or from a different source is assessed by comparing the density of triggered iPs in the period immediately preceding the CW (one refractory period, CritWin) with the period preceding CritWin (1–5 ms, RefWin). If the iP rate in CritWin was significantly smaller than in RefWin, the recording was classified as containing dependent iPs (*Dep*), otherwise to not contain dependent iPs (*No Dep*). This decision was based on the test statistic 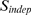 and the decision point 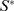 (for details see Methods).

Next, we describe the details of what constitutes a candidate potential (termed isolated potentials, iPs), how these were collected, and how statistical dependence between them and the CWs was tested. This analysis, termed Independence Assessment of Potentials (IAP), can be divided into four steps:

- *Detection of complex waveforms* (Figure 2I): All relevant potentials were collected by triggering the voltage trace at a visually determined, conservative level, i.e. to guarantee including all CWs. The waveforms of the triggered potentials were collected in the interval from 2 ms preceding to 2.5 ms following the trigger. The resulting cutouts were spike-sorted (separated into groups of similar shape) by first performing a principal component analysis (PCA) followed by a cluster-analysis (single linkage hierarchical clustering) with 4 or 5 clusters on the eigendimensions corresponding to the three largest eigenvalues. This method cleanly separates the CWs from other potentials of different shape or amplitude. Recordings with drift in the signal amplitude were excluded from analysis.
- *Detection of trigger potential* (Figure 2II): As described above, the recorded CWs contain additional components preceding the main postsynaptic component (AVCN: B, MNTB: C2). These were detected automatically within a window of 1 ms before the maximum of the main postsynaptic component by determining the local maxima in this range of the average CW. Significance of local maxima was assessed by testing (Wilcoxon signed ranks) the distribution of voltage values at the peak of the component against the distribution of the baseline fluctuations. If more than one local maximum was detected in this range, the largest was chosen for further analysis. In the AVCN this maximum corresponded to component A (see Figure 1A). In the MNTB, it corresponded to component C1 (see Figure 1B). In the case of signal transmission failures, iPs are likely to resemble the first component(s) of the CW in terms of polarity and height (AVCN: P and A, MNTB: C1). Therefore this potential, i.e. its height, served to trigger iPs in the voltage trace. For consistency across MNTB and AVCN data this potential is termed trigger potential in the following (TP).
- *Detection of isolated potentials* (Figure 2III): Before triggering iPs, the average CW was subtracted at each of the CW locations. This allowed detection of iPs that overlap with the initial and final periods of the CWs. Subtraction artifacts typically remained in the steep, central phase of the CW (see also Results on simulation data). Note, that due to these subtraction artifacts iPs overlapping with the central portion of the CW could not be detected (if present).

If the number of triggered iPs was less than 1% of the number of CWs, the recording was classified as *No Dep* since then the iPs either represent spurious voltage fluctuations, or if they were transmission failures, transmission reliability would be at 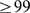
%, which can essentially be considered safe transmission.

If more iPs were triggered, they were collected in a histogram centered on the TP which gives an estimate of the temporal distribution of the iPs relative to the TP. The entries in the histogram are rescaled by 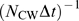 to represent the rate of iPs (in Hz) for the bin width (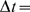
10 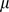
s) averaged over the number of CWs (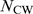) for a given recording.

IV.*Statistical comparison of AP preceding periods* (Figure 2IV): Based on this histogram and the well known biological property of a refractory period [26] on the order of 0.8–1.5 ms (depending on cell type and individual unit), it can be judged whether the iPs and the TP of the CWs stem from the same or different neuronal sources.

If they originate from different neuronal sources and are not phase locked to the stimulus (see Discussion), then the histogram should be flat, i.e. the iPs occur temporally independent with respect to the TPs of the CWs. In this case the iPs are termed independent.

If they stem from the same neuronal source, then the probability of finding iPs relative to the TPs of the CWs should ideally approach the shape of the inter-spike interval (ISI) histogram of the CWs. In this case the iPs are termed dependent (with respect to the TP of the CWs).

In the latter case the histogram will be lower within one refractory period of the TP (critical window: CritWin) than further from the TP (reference window: RefWin). Since the histogram will not always be densely sampled, a statistical test is needed that judges whether the rate of iPs in CritWin 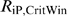 is significantly reduced in comparison to the corresponding rate in RefWin 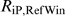. Further, this test should be balanced, i.e. not favor one of the alternatives. We also checked that the occurrence of iPs was independent of distance to the CWs, which could have influenced the histograms composition (see Results).

To account for sparsely sampled histograms, we divided RefWin into subwindows 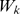 of the same length as CritWin (approx. 1 refractory period) and compared their median iP rates 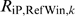 to 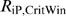 using the Wilcoxon rank sum test, each resulting in a probability 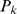 of equal medians (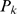 was set to 1, if 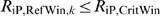, since we are only interested in rate reductions in CritWin). The final test statistic 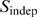 was then defined as the average of the individual probabilities for the 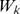


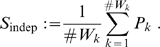



Hence, 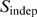 not only compares 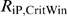 to the average of 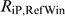 but also takes the distribution over bins in the histogram into account. This is of importance if the lack of iPs in CritWin is paralleled by periods of similar length in RefWin without iPs, i.e. 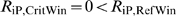 and a number of 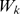 with 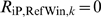. In this case, the relevance of a lack of iPs in CritWin is relativated by the lack of iPs in the 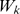.

**Figure 3:**
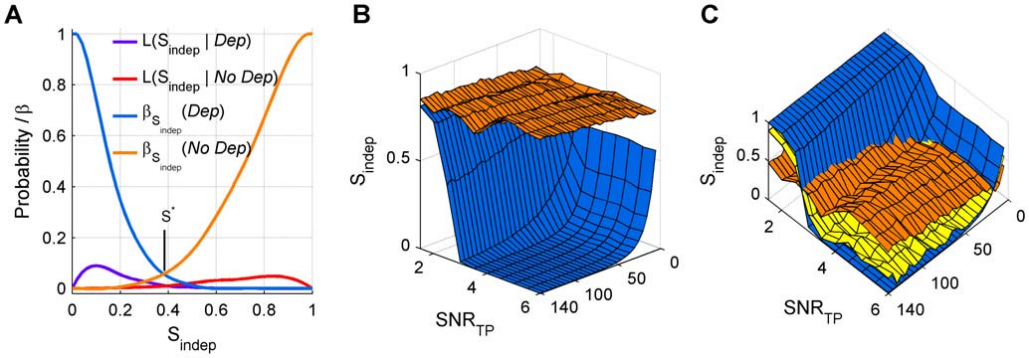
IAP decision level 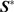. (A) 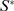 is determined by the intersection of the β-error distributions for the two cases *Dep* (blue, corresponding to failures) and *No Dep* (orange, corresponding to two units) which were generated from simulated voltage recordings. (B) Average 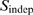 assigned by IAP to simulations with matched parameters (colors as above). On average, IAP separates *Dep* and *No Dep* for all values of the total number of iPs in the RefWin and CritWin (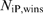) and the signal-to-noise ratio of the TP (SNR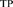). (C) One-sided 5% β-error surfaces for *Dep* and *No Dep*. The curve of intersection between these surfaces marks the parameter combinations where the common β-error drops below 5%, the criterion for including recordings. If 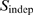 of a given recording lies below the surface of equal β-errors (yellow), it is classified as *Dep*, otherwise as *No Dep*.

While 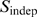 behaves similar to a probability, indicating whether to maintain the 0-hypothesis of independent iPs/no rate reduction, it is not a proper probability. Even if it were a probability, we would need to choose a significance level at which to reject the 0-hypothesis. Rather than rejecting one alternative, we determined a decision point 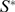 which minimizes the probability of an incorrect decision for the two hypotheses, i.e. 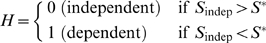
with 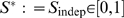 such that 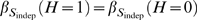, where 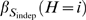 denotes the false rejection (Type II) error given that the hypothesis is true (1) or false (0) for the decision point S (see Figure 3A). The significance level of the test is then automatically upper bounded by 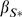. To determine 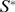 it was necessary to estimate the distributions of 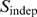 for dependent and independent iPs based on simulated voltage recordings (details are provided below).

In essence, this method implements a test for potentials approaching each other closely in time, yet with the advantage that this method also works under noisy conditions. If noise reaches the trigger level, the histogram baseline is just shifted but the difference between the number of triggers within CritWin and RefWin is preserved. Although this reduces the power of the test, it still allows a distinction between dependent and independent iPs. Further it provides a statistical test to the researcher in place of visual judgement.

### Testing and balancing the IAP

#### Simulated voltage recordings

The ability of the IAP to distinguish whether potentials stem from dependent or independent sources was validated by applying it to a range of simulated voltage traces (see Results). Here the dependence or independence of the potentials and other parameters are known and can be controlled. Briefly, the simulated voltage traces were generated by drawing spike times for a given firing rate, assigning an iP or a CW to each spike time, adding white Gaussian noise, and filtering the resulting signal similarly to the experimental apparatus. More precisely, for each stimulus condition firing rate modulations mimicking neuronal responses were generated. In the case of spontaneous firing the firing rate was assumed to be constant. In the case of the tuning stimuli we generated firing rate modulations by running a model of auditory nerve firing [27] on the experimentally used acoustic stimuli. Using these firing rates, either one (dependent) or two (independent) spike trains were drawn from an inhomogeneous Poisson process. Within these spike trains we deleted all spikes violating an absolute refractory period of 0.8 ms. In the first case the spikes in the spike train were randomly divided into iP and CW groups. In the second case one of the spike trains was assigned to the iP, the other to the CW group. Then the appropriate waveforms were positioned at the spike times from each group and summed. White Gaussian noise was added to the trace and the same bandpass filtering (0.3–7 kHz) as for the experimental data was applied to the trace. Even to the skilled eye the simulated recordings are hardly distinguishable from the real data, and thus pose a good test for the IAP method.

#### Choosing a balanced decision level

The test should distinguish between the presence (*Dep*) and the absence of dependent iPs (*No Dep*). This task differs from the classical question of whether a 0-hypothesis holds, where one specifies an acceptable level of correct classification only for the 0-hypothesis. Presently, the goal is to weigh the two hypotheses equally and choose a decision criterion that maximizes the overall percentage of correct classification.

This goal requires to minimize the Type II error of the test jointly for both hypotheses, i.e. maximize the power for both hypotheses. The power of a statistical test is usually defined as 1-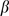, where 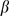 is the probability of accepting one hypothesis if it is actually false (classically the 0-hypothesis). In order to assess the power, the densities of the test statistic 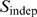 for each hypothesis, the so-called likelihoods, need to be known. We approximated the likelihoods 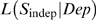 (Figure 3A, purple) and 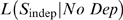 (Figure 3A, red) by subjecting simulated spike-trains of known properties to the IAP. The 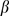-errors for the two alternatives (Figure 3A, *Dep* blue and *No Dep* orange) are minimized to the same level by choosing the point where the two functions agree. This point corresponds to the desired balanced decision point 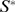. While this criterion differs from maximum likelihood, the results of the latter would have been similar since the likelihoods were always unimodal with similar tail behavior.

The considered densities depend on certain properties of the spike-train, most importantly the number of iPs in RefWin and CritWin (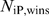), SNR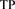, and the refractory period. Hence, also 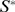 depends on these parameters. In the following, we address the dependence on SNR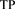 and 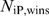, whereas for the refractory period a conservative lower limit is chosen (0.8 ms). In recordings with longer refractory periods the required minimal 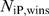 would actually be lower than indicated in the following.

From the averages of the cumulative densities (Figure 3B) it is apparent that the separation of the two hypotheses (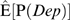, blue and 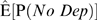, orange) depends strongly on both SNR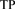 and 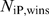. The one-sided 95% confidence bounds for both distributions, i.e. for the *Dep* case the upper and for the *No Dep* case the lower confidence bound, are shown in Figure 3C. The *Dep* (Figure 3C, blue) bound generally lies below the *No Dep* (Figure 3C, orange) bound if 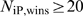 and SNR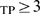. Hence, for the desired power, here 0.95, these values corresponded to the minimally required 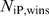 and SNR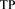. If the maximal power was lower than a desired level (0.95), we considered recordings not suited for IAP (unless iPs were scarce throughout the entire recording, see section Analysis of *No Dep* cases). If a given recording accorded to these requirements, it was classified as *Dep* if the IAP result lay below the 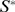 surface (yellow) and as *No Dep* otherwise.

## Results

Recordings from 55 spherical bushy cells in the AVCN and 177 principal cells of the MNTB were analyzed. If for a given unit multiple recordings existed per response condition these were concatenated unless the signal waveform or the SNR had changed in between. In the following, every unit enters into the analysis with only one (possibly concatenated) recording for each condition (if available for a given unit).

### Distribution of signal to noise ratios and firing rates

**Figure 4:**
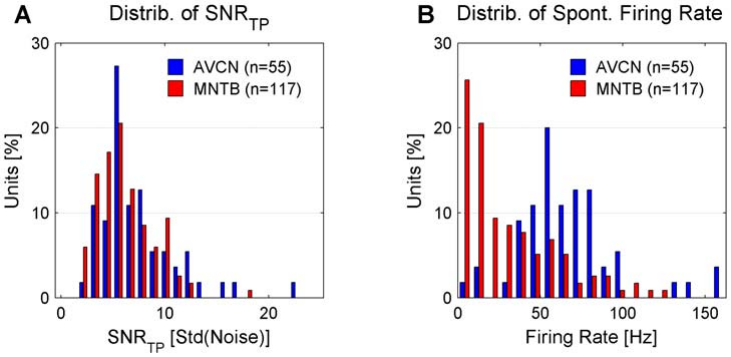
Distribution of signal to noise ratios of the TP (SNR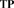) and spontaneous firing rates. (A) The distributions of SNR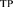 in the AVCN and MNTB are quite similar. SNR is defined as the size of a potential divided by the standard deviation of the noise (see Methods). (B) Spontaneous firing rates between the two nuclei differ in distribution with AVCN rates concentrated in the range 40–100 Hz. MNTB firing rates reach similarly high values but were concentrated below 40 Hz. 35% of MNTB firing rates exceed 30 Hz.

A statistically sound analysis of signal transmission relies on trigger potentials (TP) well above the noise level and a sufficient number of complex waveforms (CW). Extracellular recordings from both nuclei studied exhibited a wide range of signal to noise ratios of the TP (SNR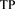) and firing rates (Figure 4). Recordings were further analyzed if the SNR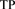 exceeded 3 and more than 200 CWs could be collected. For the AVCN 130 (53 spontaneous, 31 excitatory, 46 inhibitory/suppressive) and for the MNTB 241 (89 spontaneous, 65 excitatory, 87 inhibitory/suppressive) recordings passed these criteria. IAP analysis could be applied at a 5% error level for 121 AVCN (48 spontaneous, 30 excitatory, 43 inhibitory/suppressive) and for 189 MNTB (66 spontaneous, 57 excitatory, 66 inhibitory/suppressive) recordings.

### Representative examples of IAP analysis

The IAP provides a statistical tool to distinguish whether different potentials were emitted by a single neuronal source or multiple independent neuronal sources. If a single source emitted the potentials, their temporal relationship would be influenced by the refractory period. If they were emitted by multiple, independent sources, their temporal relationship should be unrestricted. Analyzing this relationship in terms of the rate of iPs as a function of interpotential distance, rather than a distance criterion for pairs of potentials, allows us to extend the analysis to conditions of noise and comparably low SNR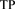. These conditions are common for extracellular recordings *in vivo* and have to be taken into account if synaptic transmission is studied.

#### Simulation

**Figure 5:**
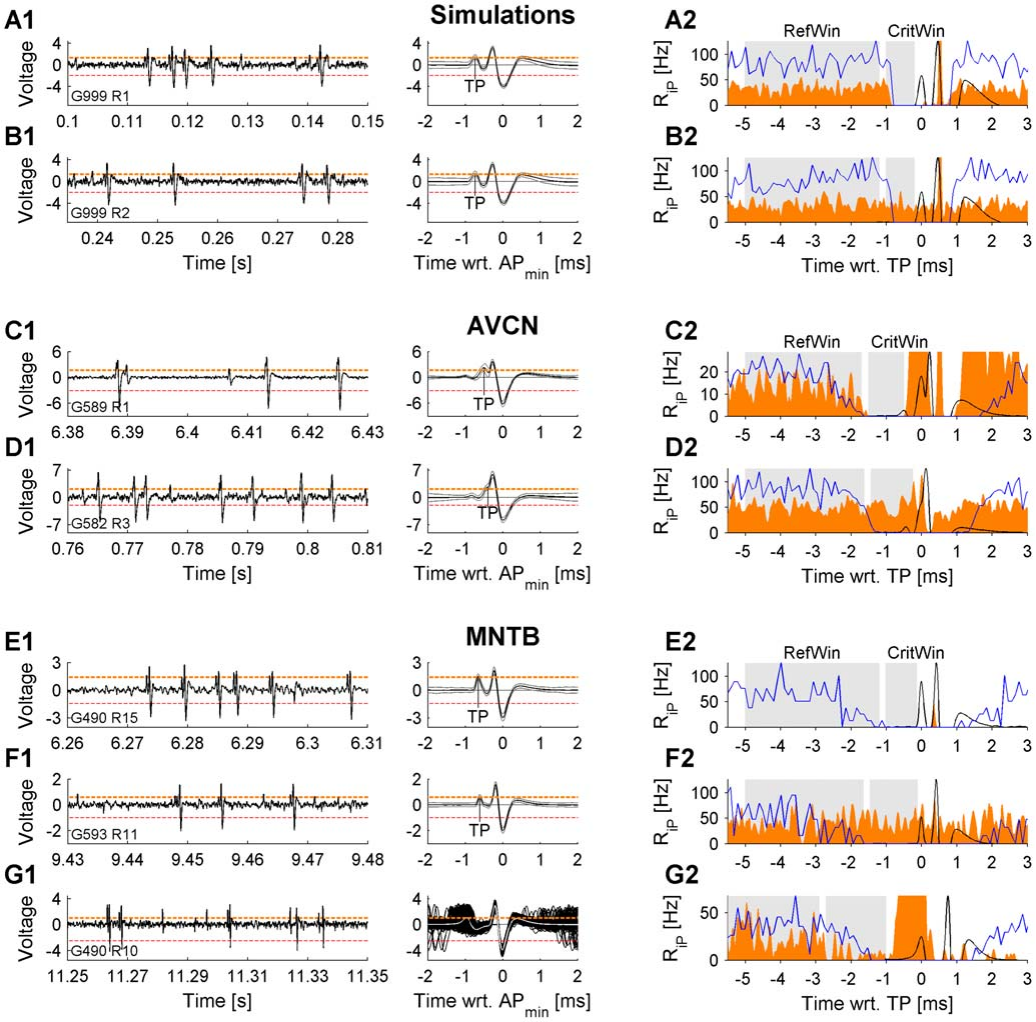
Examples of IAP for simulated, AVCN, and MNTB recordings of spontaneous activity. The left column shows voltage traces (black) and trigger levels for complex (red) and candidate waveforms (orange) for each unit. The corresponding average complex waveform (black) and its pointwise standard deviation (gray) is depicted in the middle column. The trigger potential (TP) (MNTB: presynaptic spike, AVCN: postsynaptic potential, probably the EPSC) is also indicated. The right column shows the histograms (orange) generated by triggering at the height of the TP (after aligned subtraction of the average complex waveform). Further the interspike interval histograms of the complex waveforms are shown, mainly for visual comparison. For the simulated data (A2, B2) the histograms reflect the failure containing condition by a decrease in CritWin (A2), and conversely the lack of decrease in the two unit condition (B2). Guided by the results from the known datasets, the AVCN data (C2, D2) can be interpreted: A substantial number of cells exhibited histograms similar to the cell in C2, suggesting failures of transmission, while the remaining cells showed histograms similar to the cell in D2. If a decrease occurred, its timing was predicted by the ISI histogram (blue). In the MNTB (E2, F2, G2) the most frequent finding was the absence of iP at the TPs height, leading to an empty histogram as in E2. Most of the units with iPs of sufficient height, exhibited no decrease of the histogram in CritWin, as in F2. In a small fraction of recordings a decrease was observed, yet, this could be accompanied by unusually high variability in timing from the presynaptic to the postsynaptic side as in G2 (see individual trace in middle column). IAP classified the recordings in A,B, and G as *Dep* and the remaining as *no Dep*.

The following representative results for a *Dep* and a *No Dep* simulation serve to provide intuition for the subsequent interpretation of IAP results of experimental data where the exact dependence between CWs and iPs is unknown (Figure 5A, B). We created simulated data sets closely mimicking voltage traces of AVCN/MNTB units. In these data sets, all parameters can be controlled, especially the dependence between CWs and iPs. In both examples, the CW and the iP firing rates were constant at 50 Hz and periods of 100 s were simulated.

For the *Dep* simulation the iP histogram shows the expected decrease in iP rate in CritWin (Figure 5A2). Qualitatively, the iP rate follows the ISI histogram of the CWs which is a consequence of both potentials originating from the same source. For the *No Dep* simulation, the iP rate remains unchanged in CritWin (Figure 5B2). The IAP significance assessment correctly classified both examples as *Dep* and *No Dep*, respectively.

As briefly noted in Methods, the subtraction of the mean CW from each CW leaves subtraction artifacts in the steepest phases of the CW, often including the fast positive and negative portions of the postsynaptic AP (see Figure 5A2/B2). Since these artifacts were also observed in *Dep* simulation data, i.e. where iPs are known not to overlap with CWs, these phases of the CW were not included in the IAP analysis. Note, if perfect subtraction had been possible, CritWin could have been chosen symmetrical around the TP, thus enlarging the statistical basis.

#### AVCN

Both *Dep* and *No Dep* results were frequently obtained for recordings of AVCN units. A recording of spontaneous activity leading to a *Dep* result is depicted in Figure 5C (CF 1.3 kHz). The iP rate decreased strongly in CritWin, characteristic for potentials from a single source separated by a refractory period. As in the simulated data, the iP rate preceding the TP qualitatively follows the ISI histogram of the CWs. As shown in this example, *Dep* results were often accompanied by asymmetrically distributed (with respect to the TP) iP rates with higher rates following the TP/CW than preceding it. In contrast, the recording depicted in Figure 5D (CF 1.29 kHz) neither shows a decrease of iP rate in CritWin nor an asymmetric distribution of the iP rate. Although the difference between the two recordings is obvious from their iP histograms, it is hardly noticeable from their voltage traces.

#### MNTB

In the MNTB, almost all recordings were classified as *No Dep*, mostly because of the absence of iPs throughout the whole recording. An example of this kind, in this case a recording of spontaneous activity, is shown in Figure 5E (CF 11.4 kHz). Isolated potentials are triggered neither in CritWin nor in RefWin. Most of the recordings that contained iPs resulted in flat iP rate histograms, i.e. no significant reduction of the iP rate in CritWin. Results for such a recording of spontaneous activity are shown in Figure 5F (CF 3.9 kHz). As in the *No Dep* case of the AVCN, no asymmetry of iP rates is observed. In Figure 5G one of the few MNTB recordings is shown (CF 16.0 kHz) which met the criteria for *Dep* classification. While this demonstrates that IAP can detect (putative) failures in the MNTB as well, the interpretation of this particular case is involved. In contrast to the great majority this unit exhibited an unusually high variability in the delay from C1 to C2 (0.5 ms–2 ms, see superimposed waveforms in Figure 5G1 right), i.e. probably between the presynaptic and the postsynaptic AP. This variability might be an indication of somehow modified transmission, e.g. through modified cellular dynamics on the postsynaptic side or modified transmission at the synapse. Also, injury by the microelectrode cannot be excluded. Since this variability only occurred in two cases, we could not develop a good interpretation for these, probably not representative recordings.

Voltage traces, average waveforms, and iP rate histograms showed a similar behavior under excitatory and inhibitory/suppressed response conditions. We verified that the IAP results remained accurate under conditions of variable response rates (data not shown).

### IAP population analysis

**Figure 6:**
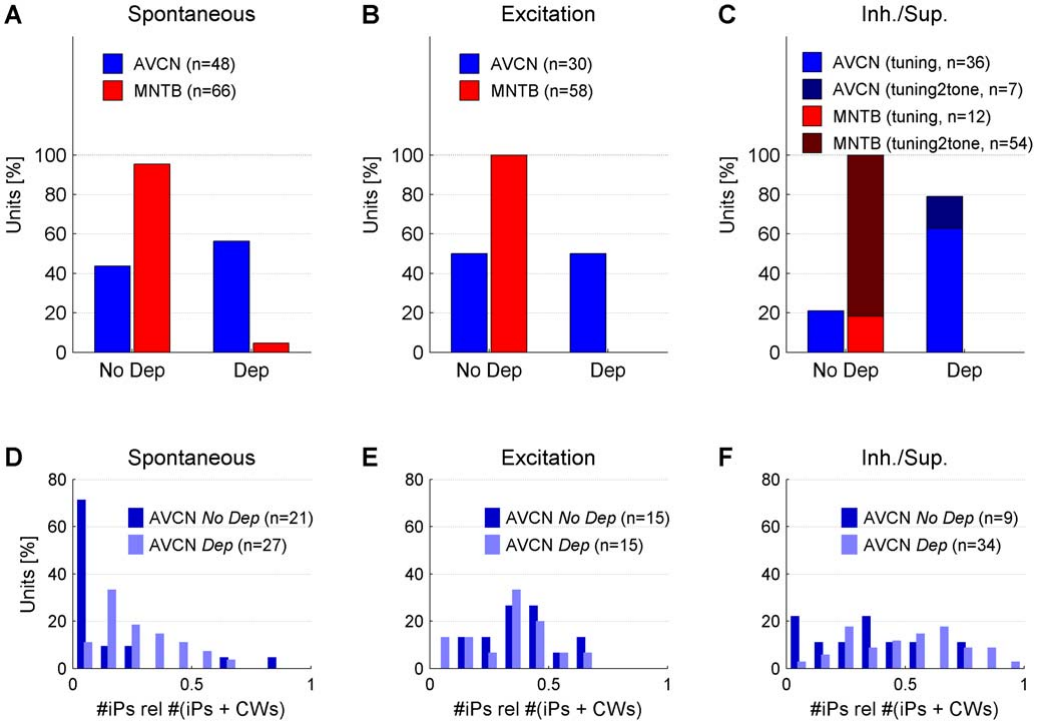
IAP results and proportion of iPs for AVCN and MNTB for each recording condition. (A) For spontaneous activity the majority (56%) of the AVCN, but less than 5% of the MNTB recordings contained dependent iPs. (B) In the excitatory response condition the percentage of AVCN *Dep* recordings decreased to 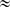
50%. In the MNTB no *Dep* recordings were found. (C) In the inhibitory/suppressive response condition the percentage of AVCN *Dep* recordings increased to 80%, whereas again none of the MNTB recordings was classified as *Dep*. For AVCN *Dep* recordings the proportion of iPs with respect to the total number of (presynaptic) events estimates the insecurity of transmission (if iPs correspond to failures of transmission). (D) During spontaneous activity *No Dep* had a significantly smaller iP proportion than *Dep*, consistent with failures. (E) In the excitatory condition the iP proportion does not differ significantly between the *No Dep* and the *Dep* cases, although they both increase compared to the spontaneous condition. (F) In the inh./sup. condition the distribution of iP proportion for the *Dep* cases increases significantly compared to both previous conditions, whereas the corresponding distribution for the *No Dep* cases stays similar to the excitatory condition.

Across all recording conditions, dependent iPs were frequently found in the AVCN, but rather rarely in the MNTB. In the case of spontaneous activity, 56% (27/48) of the AVCN recordings yielded a *Dep* result (Figure 6A). For the MNTB, 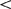
5% (3/66) of the recordings were classified as *Dep*.

In the excitatory response condition, the percentages of the *Dep* result were reduced. In the AVCN, the percentage dropped to 50% (15/30), in the MNTB no recordings classified as *Dep* remained (Figure 6B). These reductions were likely to be caused by a significant increase (+20%) in standard deviation of the baseline voltage fluctuations (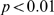, Wilcoxon signed rank test for matched samples, 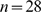), probably due to the activation of neighboring cells.

**Figure 7:**
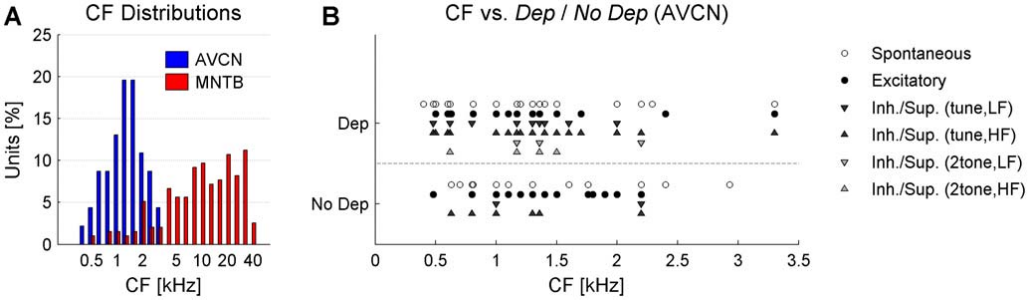
*Dep* recordings are not correlated with CF. (A) The distributions of CF for both nuclei differ: AVCN units (blue) are concentrated in the low frequency range reaching up to 3 kHz. The MNTB CFs (red) range over the whole spectrum, yet typically exceeding 3 kHz. The overlap of the MNTB with the AVCN range amounts to 16%. (B) Distribution of *Dep* and *No Dep* recordings in the AVCN with respect to CF for all recording conditions (see legend). If the classification as *Dep* was correlated with lower CFs (where stronger phase-locking occurs), the average CF of the *Dep* cases should be significantly lower than the average CF of the *No Dep* cases. The statistical comparison was not significant in any of the conditions (spontaneous, excitatory, single [black] and two-tone [gray] stimulations in the low- [LF] and high-frequency [HF] inhibitory/suppressive response regions) with all 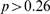 (Wilcoxon rank sum test for different medians of two groups).

In the inhibitory/suppressive response condition (Figure 6C), dependent iPs are expected to occur as failures of signal transmission as demonstrated in previous studies (pharmacologically in the AVCN [12] and by means of distinguishing pre- and postsynaptic potentials in the MNTB [14]). Indeed, in the AVCN the IAP yielded an increased percentage of *Dep* recordings (79%, 34/43). In contrast, no MNTB recording was classified as *Dep*. Interestingly, in the AVCN the standard deviation of the baseline voltage during this response condition was not significantly increased (+2%, 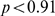, Wilcoxon signed rank test for matched samples, 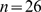) compared to the spontaneous condition. Note, that the different proportions of single and two-tone stimulations between AVCN and MNTB are a consequence of the lower spontaneous rates in MNTB units (Figure 4), more often requiring the use of a two-tone paradigm. Results from low frequency and high frequency inhibitory fields have been pooled in the histogram to avoid crowding the figure (see Figure 7B for separated low frequency and high frequency results).

The classification of the AVCN units across recording conditions was quite consistent: All units that were classified as *Dep* in the spontaneous and/or the excitatory condition were also classified as *Dep* in their suppressive/inhibitory condition. Between the spontaneous and the excitatory condition the agreement was 87% for the *Dep* cases.

### Proportion of iPs with respect to all potentials

Whether a recording is classified as *Dep* or *No Dep* does not determine the abundance of iPs. In the *Dep* cases the abundance of iPs indicates the failure rate of the synapse if the iPs actually correspond to failures of transmission (see Discussion). More precisely, the failure rate would be estimated by the proportion of iPs with respect to the total number of iPs and CWs. Assuming that some of the iPs are due to noise of other cells, this proportion provides an upper bound on the failure rate. For comparison, we also provide this proportion for the *No Dep* cases, although here, it cannot be interpreted as a failure rate. Since the data of the three (spontaneous) MNTB cases classified as *Dep* (iP proportion: 0.21, 0.41, 0.57) do not provide sufficient information for a histogram, only the AVCN data are considered (Figure 6).

For the recordings of spontaneous activity, the distributions of iP proportion for *Dep* and *No Dep* differ significantly in their medians (0.24 SD 0.17, 0.11 SD 0.23, Wilcoxon rank sum test for equal medians, 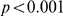, Figure 6D). In the *No Dep* recordings the values are concentrated below 0.1, whereas in the *Dep* recordings they are broadly distributed up to 0.7. For the excitatory condition both distributions shift to higher values (Figure 6E). For the *No Dep* recordings the mean increased significantly (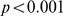), probably reflecting the activation of neighboring cells. The shapes and means of the distributions are very similar, i.e. no significant difference exists between them (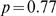). For the inhibitory/suppressive condition the *Dep* distribution broadens/shifts significantly to a higher mean value (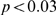), whereas the *No Dep* distribution retains a similar mean (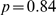, Figure 6F). Although the mean of *Dep* (0.48 SD 0.19) exceeds the *No Dep* (0.36 SD 0.24) mean, the variability seen in the data prevents a statement of significant difference in mean. Generally, the iP proportion is quite variable for both *Dep* and *No Dep* with the exception of spontaneous *No Dep* recordings. This is consistent with previous findings [12].

It might not be surprising that the iP proportion for the *No Dep* cases is also high in the excited and suppressive/inhibitory conditions, since neighboring cells are likely to have similar activity levels under the same stimulation.

### IAP results are not correlated with CF

The contrasting IAP results between AVCN and MNTB units could be related to their different distributions of CF. The CFs of AVCN units ranged from 0.3 to 3.3 kHz, CFs of MNTB units from 0.5 to 45 kHz (Figure 7A). In the frequency range up to approx. 2 kHz, both AVCN and MNTB units phase-lock to the sinusoidal stimulus [28]–[30]. If the iPs also phase-lock, TPs and iPs would tend to be separated by an interval corresponding to the frequency of phase-locking. Using the IAP could result in the interval being interpreted as the refractory period, thus classifying the iPs as *Dep* even if they actually originate from different sources. Although this classification is correct in the sense that both potentials depend on the stimulus, it would invalidate the study of signal transmission. Therefore, we addressed this concern by comparing the CFs of *Dep* and *No Dep* recordings separately for each condition (Figure 7B). In the AVCN, no significant differences in mean CF between *Dep* and *No Dep* results were found (Wilcoxon rank sum test for two groups, spontaneous 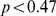; excitatory 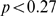; inhibitory/suppressive: tuning LF 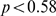, tuning HF 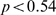, only *Dep* cases for two-tone tuning). In the MNTB, the distribution of CFs has most of its weight above 3.5 kHz (Figure 7A) corresponding to the distribution of CFs in the MNTB across species [31], [32]. Assuming that phase-locking occurs up to approx. 2.5 kHz in the afferents of the MNTB [29], only one of the *Dep* cases from spontaneous recordings had a CF in the relevant range (CFs: 2.4, 4.0, and 16.0 kHz).

### Longer stimuli do not lead to more *Dep* cases in the MNTB

In the excitatory condition a given unit usually emits thousands of CWs (mean: 2600 SD 1378) within approximately 60 s (depending on the number of repetitions), leading to an average firing rate of 80 Hz (SD 37 Hz). Due to the nature of auditory tuning curves in the MNTB, the response consists of episodes of very high firing rate (up to 500 Hz over 100 ms) alternating with periods of little activity.

To test whether failures (and consequently *Dep* cases) occur at the calyx of Held only for longer periods of more intense activity, another excitatory response condition was tested. A broadband, noise-like stimulus (reaching from two octaves below CF to one octave above CF) was presented for 100 s almost continuously (100 ms pause every 5 s due to technical limitations). The loudness of the noise was adjusted to obtain a high firing rate (132 SD 38 Hz). This paradigm was employed in 22 cells with a high SNR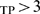. However, the results qualitatively agree with the other conditions in the MNTB. Two units were classified as *Dep*, suggesting only a small increase in *Dep* cases due to the prolonged, higher frequency discharge.

### Windows of analysis provide representative iP counts

The judgement of the presence of dependent iPs with the IAP is based only on the iPs that fall into the windows preceding the APs (RefWin/CritWin). It is hence important to assess, whether the collection of iPs in the windows is representative for the iPs in the whole recording.

**Figure 8:**
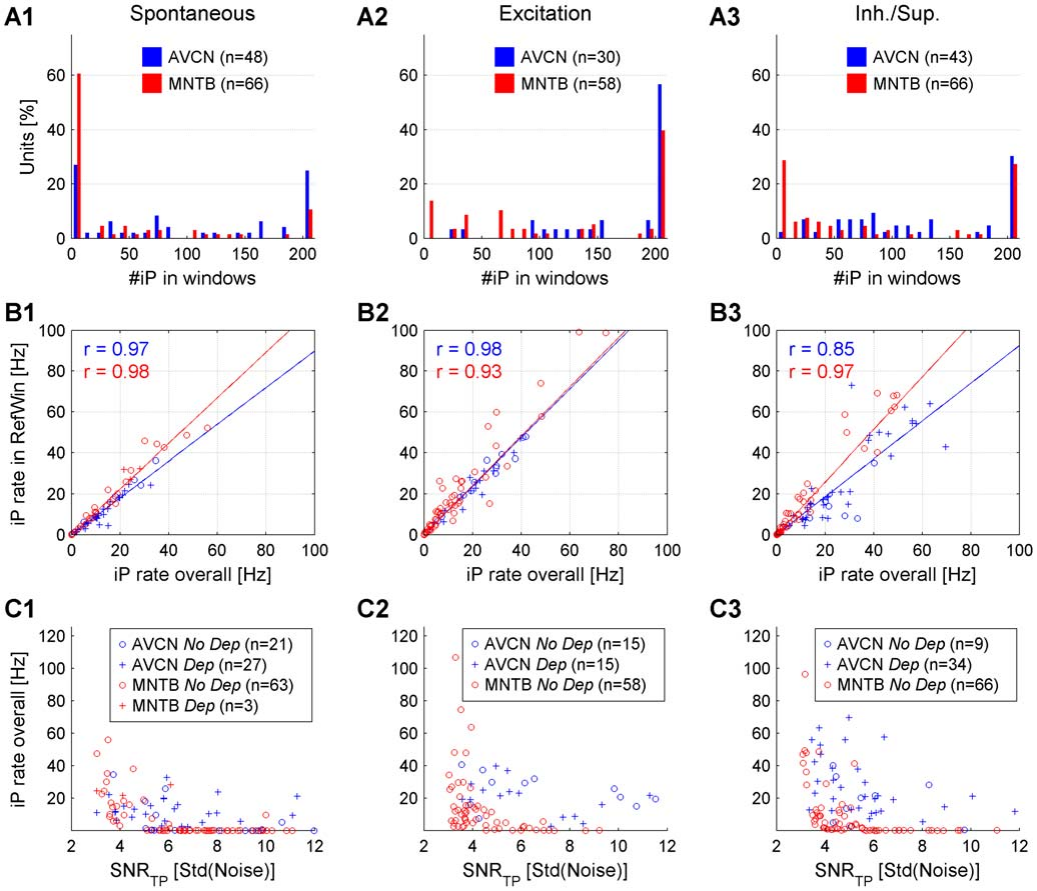
Statistics and correlations for the number of iPs and different iP rates. (A1–3) #iPs in the two windows (RefWin,CritWin) for all recording conditions. In the AVCN iPs are generally more abundant, reflected in higher #iPs in the windows. In the MNTB the #iPs in the windows rises in the excited condition, probably due to activation of neighboring units. In the inh./sup. condition the #iPs in the windows is again reduced, probably due to correlated inh./sup. areas for neighboring units. (B1–3) The iP rate in RefWin equals or exceeds the overall iP rate, thus confirming that the IAP is not biased by considering only iPs close to the CWs. Especially for the MNTB, low iP rates in RefWin entail similarly low overall iP rates, hence correspond to very low #iPs rel. to #CWs. (C1–3) A comparison of SNR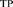 and the overall iP rate between the two nuclei shows that for the best SNR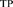, in the AVCN the overall iP rate often remains substantial, whereas it drops to vanishingly low values in the MNTB. This indicates that in the MNTB virtually no iPs remain under conditions where dependent iPs should be best detectable.

In both AVCN and MNTB, a considerable percentage of the recordings contained only few or no iPs in RefWin/CritWin, most prominent in spontaneous recordings from the MNTB, where approx. 70% had less than 10 iPs in RefWin/CritWin corresponding to low iP rates in the windows (Figure 8A1–A3). Are these low iP rates in agreement with the iP rates observed in the whole recording, or do iPs dominantly occur at a greater temporal distance to the APs? In this comparison only the iP rate in RefWin can be used, as the iP rate in CritWin would be reduced by the refractory period between the TPs and the dependent iPs, if present.

In all response conditions (spontaneous, excitatory, inhibitory/suppressive) the rate of iPs in RefWin was a good predictor of the total iP rate (Figure 8B1–3, AVCN: blue, MNTB: red, *No Dep*: circles, *Dep*: pluses). This is indicated by the high r values together with slopes close to 1. In some cases the iP rate in RefWin even exceeded the overall iP rate, indicated by slopes greater than 1. This can be explained, assuming that all potentials, APs and iPs, are more likely to be emitted during stimulation than during silent periods. Note that low rates of iPs inside RefWin correlate extraordinarily well with low overall rates. Hence, the recordings with few or no iPs in RefWin/CritWin were correctly judged as *No Dep* since neither dependent nor independent iPs occurred in relevant numbers in the whole recording.

### Relation of SNR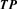 and iP rate consistent with the IAP results

Finally, we compare the SNR of the trigger potential (SNR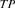) to the rate of iPs in the whole recording (Figure 8C1–3). Recordings with highest SNR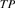 should provide the best conditions for detecting failures of transmission, since then TPs and their isolated counterparts should be most easily detectable. For the AVCN (blue circles and pluses), many recordings for all three conditions exhibited high SNR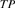 and considerable rates of iPs, a considerable percentage of which were judged as dependent (blue pluses). In the MNTB (red circles and pluses), recordings with high SNR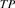 typically had very low rates of iPs. For both AVCN and MNTB the larger rate of iPs at low SNR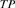 are most probably contributed by triggering baseline fluctuations of similar size as the TP rather than triggering iPs.

## Discussion

The present study investigated the reliability of transmission at the giant synapses of Held *in vivo* using a novel statistical method. Our analysis provides further evidence consistent with previous findings on signal transmission at the endbulbs of Held (AVCN) while indicating the need for reconsideration in the case of the calyces of Held (MNTB). In the MNTB only few recordings contained dependent iPs, thus rendering isolated prepotentials, i.e. failures of signal transmission, a rare event. By contrast, in the AVCN the majority of recordings contained dependent iPs suggesting failures of AP transmission, predominantly during the inhibitory/suppressive response conditions.

The different results at the two synapses of Held emphasize the necessity for systematically assessing the origin of failure candidates before assuming a common origin with the complex waveforms. The decision as to whether potentials in a recording are dependent or not has to be based on extended stretches of the recording. Only a statistical approach can provide a reliable decision based on the probability with which the events occur in relation to the complex waveforms.

### Origin of dependent iPs

In principle, a number of different constellations can lead to the observed dependence between iPs and complex waveforms. This ambiguity necessitated the present cautious choice of terminology, i.e. dependent iPs rather than failures. Based on the current biological understanding of the investigated nuclei, we will argue below that dependent iPs do most likely correspond to failures of synaptic transmission. Since dependent iPs were rare in the MNTB, we will focus on the AVCN.

Aside from failures, a common cause can lead to dependence between iPs and the complex waveforms of a given unit: Most directly, dependence would be induced, if the iPs originate from a source which receives input via the same auditory nerve fiber. In this case, the second source would be another spherical bushy cell. Alternatively, dependence would also be present, if the given unit and the iPs are phase-locked to the same stimulus, which would also synchronize their refractory periods. Here, the second source could either be another spherical bushy cell or another auditory nerve fiber.

The tuning of the iPs has been shown to be V-shaped and monotonic, whereas the tuning of the complex waveforms is often non-monotonic at the CF [12]. This renders other spherical bushy cells unlikely as iP sources and leaves another, phase-locked auditory nerve fiber as a possible source. However, the present results are not consistent with the hypothesis that phase-locking plays a major role in IAP classification: (i) Classification into *Dep* and *No Dep* was not CF dependent (as detailed in Results). (ii) The duration of CritWin was not correlated with the period corresponding to the unit's CF. This would have been expected if it arose from phase-locking. (iii) The percentage of *Dep* results in the excited condition was lower than for spontaneous recordings, contrary to the expectation that during excitation stronger phase-locking would have increased the number of *Dep* cases.

Further lines of evidence based on the waveforms have been provided [33], but may not be necessary. Taking these arguments together with previous accounts for failures in the AVCN (see below) provides convincing evidence for assuming that dependent iP constitute failures at the endbulbs of Held *in vivo*.

### Endbulbs of Held - AVCN

The size of the endbulbs of Held and their involvement in auditory processing at high temporal precision rendered them a possible candidate for reliable signal transmission. However, consistent with the present study, several earlier studies have demonstrated that failures of transmission occur both *in vitro* ([15], especially for stimulation rates exceeding 100 Hz) and *in vivo* [11], [12]. While synaptic depression has been suggested as a major cause [15], glycinergic and GABAergic inhibition could also induce failures. Both anatomical [34]–[38] and physiological [12], [39]–[42] findings provided evidence for a role of these inhibitory transmitters in modulating synaptic transmission, e.g. in response to certain stimulus configurations. The present results add to the evidence for the presence of inhibition, since synaptic depression does not predict an increase in transmission failures in the case of sound evoked suppression/inhibition. To evaluate the relevance of inhibition, the possibility of interaction with different anesthetics would have to be checked.

Quantitatively, the present percentage of recordings showing failures (40–70%, depending on recording condition) differs from both the estimates in [11] (20–25%) and [12] (50–100%, 21/42 units with complex waveforms and 21/21 units studied with waveform analysis). While the estimation method is not clearly stated in [11], the reduction in comparison to [12] can be explained by the present distinction of dependent from independent iPs.

Functionally, the occurrence of failures of transmission does not necessarily imply imprecision, but can rather be a means of temporal sharpening. Phase-locking to pure tones has been shown to improve from auditory nerve fibers to the spherical bushy cells [28], [43], which are connected via the endbulbs of Held. Different mechanisms could contribute to this improvement: First, precisely timed inhibition could prevent imprecise spikes. Second, since usually multiple endbulbs converge on a single spherical bushy cell [3], [4], [44], the improvement in phase-locking could be due to the detection of coincident inputs [45], [46]. Interestingly, for coincidences to become relevant, synaptic depression is a necessary ingredient, especially after the temporally more precise onset response. In both mechanisms, certain, probably poorly phase-locked endbulb inputs will not elicit postsynaptic spikes thus leading to failures.

### Calyx of Held - MNTB

Based on its elaborate morphology and a number of *in vitro* studies, the calyx of Held was long thought to provide reliable signal transmission [5], [17], [47]. Similar to the AVCN, synaptic depression was shown to reduce the safety factor, yet leaving AP transmission intact, especially after hearing onset [17], [18]. Though principal cells of the MNTB had earlier been shown to be contacted by inhibitory synaptic terminals [48]–[51], the sensitivity of MNTB principal cells to glycine and GABA has only recently been demonstrated *in vitro* [52]–[54].

Along these lines, failures of transmission at the calyx of Held were found in only three studies. In the cat failures were described *in vivo* under electrical stimulation at high frequencies (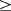
500 Hz, [13]). Yet, for sound evoked responses tested, these authors “searched extensively, but did not find inhibition which blocked C2 following C1” (p. 329, C1: presynaptic, C2: postsynaptic). The waveform analysis conducted in a later study in the gerbil [14] showed failures in sound evoked responses for 35% (30/85) of the units. In two recent *in vitro* studies failures occurred during high-frequency electrical stimulation in synapses after prolonged conditioning with high firing rates (gerbil: [19], mouse: [20]). Only limited agreement exists between these reports and the present study, as transmission failures were observed only rarely during spontaneous responses and in only a small fraction of recordings after prolonged acoustic stimulation.

Focusing on acoustic stimulation *in vivo*, the present findings qualitatively mirror the observations in [13] and [21]. The difference to [14] results from the IAP criterion, which classified most iPs as independent. The reason for failures following electrical stimulation, however, remains elusive, especially as average firing rates during acoustic stimulation usually match rates used in electrical stimulation (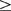
300 Hz). Since Guinan et al. [13] observed this discrepancy between electrical and acoustical stimulation in very similar preparations, differences due to recording and analysis techniques are unlikely for this study in particular.

However, it has recently been proposed that activity induces increases in the level of nitric oxide which in turn reduce the excitability of MNTB neurons and can lead to failures of signal transmission [20] (*in vivo* after 10–30 min of 80 dB stimulation at CF under fentanyl anesthesia). Since ketamine, the anesthetic commonly used in *in vivo* experiments, interferes with the production of nitric oxide one might propose that failures are more commonly observed in awake animals which are exposed to extended periods of acoustic stimulation. Until further exploration of this issue we would conclude that failures of transmission are rare at the calyx of Held at least under the kind of acoustic stimulation, anesthesia, and species investigated in the present and the former *in vivo* studies [13], [21]. If failures prove to be rare at the calyx of Held, the MNTB's function might actually be the previously proposed “sign inversion”.

### Independence assessment of potentials

Although IAP was designed merely as an automated and statistically grounded procedure for spike-sorting and temporally comparing certain potentials, some discussion concerning its underlying assumptions, performance limits, and dependence on the shape of CW are provided.

#### Assumptions

IAP relies on only few assumptions:

- Potentials transmitted via the same fiber are assumed to obey a refractory period. The duration of the refractory period was not critical for the performance of IAP and inter-spike intervals 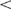
0.75 ms were not observed.
- AP waveforms are assumed not to exhibit systematic variations in shape or size over the recording period (e.g. caused by the animals breathing, electrode dislocation, etc.). This requirement was checked visually for each recording prior to analysis.
- Dependent iPs are assumed to be of similar size as their corresponding TPs in CWs. This assumption bears the greatest biological relevance since its validity is determined by the cause of the dependent iPs. In the MNTB sizes are known to be similar at least under electrical stimulation [13], [19], [24]. In the AVCN many (including the present) studies argue in favor of this assumption. Obviously, further failures of a different kind, violating the size assumption, cannot be excluded, yet we have re-run the analysis with iPs triggered at only 50–70% of the TP height with qualitatively identical results (not shown).
- Potentials from different sources are assumed to not be closely correlated in time. Phase-locking could have invalidated this assumption, but apparently did not play a significant role (see Results). Note, however, that this question only has a minor influence for MNTB data, as the CFs predominantly lie above 2.5 kHz [29].

#### Performance limits

Aside from a small number of CWs, IAP performance is mainly limited by the ratio between dependent and independent iPs/noise. If the rate of dependent iPs is far lower than the rate of independent iPs, the reduction in CritWin will only become significant after very long recording periods containing large numbers of CWs. Yet, it is debatable whether very low failure rates are of interest for the study of transmission reliability.

#### Shape of complex waveform

The shape differences of the CWs recorded in AVCN and MNTB could bias the results obtained in each nucleus (Figure 1). The CWs mainly differ in two respects: Firstly, in the AVCN two components precede the postsynaptic AP, whereas only one in the MNTB. Secondly, in the AVCN the larger component (A, used as TP) is closer to the postsynaptic AP (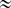
0.1 ms) than C1 is to the postsynaptic component in the MNTB (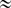
0.5 ms). Using simulations based on each CW separately we could confirm that the classification by IAP remains valid independent of the CW (data not shown). Concerning the second difference no bias would be expected since the temporal separation between A/C1 and the postsynaptic does not influence the histogram before A/C1. The first difference (additional component P in the AVCN) could have had an influence on the histogram since it could have contributed to the histogram in CritWin, thus rendering *Dep* classification less likely. As mentioned in Methods we accounted for this effect by subtracting the average CW from each CW. Also, only a small effect would be expected since P's height is usually only about 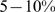 of A's height.

## References

[1] Schneggenburger R, Forsythe ID (2006). The calyx of Held.. Cell Tissue Res.

[2] Held H, His W, Bois-Reymond ED (1893). Die zentrale Gehörleitung.. Archiv für Anatomie und Physiologie.

[3] Ryugo DK, Fekete DM (1982). Morphology of primary axosomatic endings in the anteroventral cochlear nucleus of the cat: a study of the endbulbs of Held.. J Comp Neurol.

[4] Sento S, Ryugo DK (1989). Endbulbs of Held and spherical bushy cells in cats: morphological correlates with physiological properties.. J Comp Neurol.

[5] Morest DK (1968). The growth of synaptic endings in the mammalian brain: a study of the calyces of the trapezoid body.. Zeit Anat EntwGesch.

[6] Sivaramakrishnan S, Laurent G (1995). Pharmacological characterization of presynaptic calcium currents underlying glutamatergic transmission in the avian auditory brainstem.. J Neurosci.

[7] Forsythe ID (1994). Direct patch recording from identified presynaptic terminals mediating glutamatergic EPSCs in the rat CNS, *in vitro*.. J Physiol (Lond).

[8] Barnes-Davies M, Forsythe ID (1995). Pre- and postsynaptic glutamate receptors at a giant excitatory synapse in rat auditory brainstem slices.. J Physiol (Lond).

[9] Borst JGG, Helmchen F, Sakmann B (1995). Pre- and postsynaptic whole-cell recordings in the medial nucleus of the trapezoid body of the rat.. J Physiol (Lond).

[10] Takahashi T, Forsythe ID, Tsujimoto T, Barnes-Davies M, Onodera K (1996). Presynaptic calcium current modulation by a metabotropic glutamate receptor.. Science.

[11] Pfeiffer R (1966). Anteroventral Cochlear Nucleus: Wave Forms of Extracellularly Recorded Spike Potentials.. Science.

[12] Kopp-Scheinpflug C, Dehmel S, Dörrscheidt GJ, Rübsamen R (2002). Interaction of excitation and inhibition in anteroventral cochlear nucleus neurons that receive large endbulb synaptic endings.. J Neurosci.

[13] Guinan JJ, Li RY (1990). Signal processing in brainstem auditory neurons which receive giant endings (calyces of Held) in the medial nucleus of the trapezoid body of the cat.. Hear Res.

[14] Kopp-Scheinpflug C, Lippe WR, Dörrscheidt GJ, Rübsamen R (2003). The medial nucleus of the trapezoid body in the gerbil is more than a relay: comparison of pre- and postsynaptic activity.. J Assoc Res Otolaryngol.

[15] Wang Y, Manis PB (2006). Temporal coding by cochlear nucleus bushy cells in DBA/2J mice with early onset hearing loss.. J Assoc Res Otolaryngol.

[16] Chuhma N, Ohmori H (1998). Postnatal development of phase-locked high-fidelity synaptic transmission in the medial nucleus of the trapezoid body of the rat.. J Neurosci.

[17] Taschenberger H, von Gersdorff H (2000). Fine-tuning an auditory synapse for speed and fidelity: developmental changes in presynaptic waveform, EPSC kinetics, and synaptic plasticity.. J Neurosci.

[18] Futai K, Okada M, Matsuyama K, Takahashi T (2001). High-fidelity transmission acquired via a developmental decrease in NMDA receptor expression at an auditory synapse.. J Neurosci.

[19] Hermann J, Pecka M, von Gersdorff H, Grothe B, Klug A (2007). Synaptic transmission at the calyx of Held under *in vivo* like activity levels.. J Neurophysiol.

[20] Steinert JR, Kopp-Scheinpflug C, Baker C, Challiss RAJ, Mistry R (2008). Nitric oxide is a volume transmitter regulating postsynaptic excitability at a glutamatergic synapse.. Neuron.

[21] Mc Laughlin M, van der Heijden M, Joris PX (2008). How secure is *in vivo* synaptic transmission at the calyx of Held?. J Neurosci.

[22] Tolnai S, Englitz B, Kopp-Scheinpflug C, Dehmel S, Jost J (2008). Dynamic coupling of excitatory and inhibitory responses in the medial nucleus of the trapezoid body.. Eur J Neurosci.

[23] Guinan JJ, Guinan SS, Norris BE (1972). Single auditory units in superior olivary complex 1. responses to sounds and classifications based on physiological properties.. Int J Neurosci.

[24] Wu SH, Kelly JB (1992). Synaptic pharmacology of the superior olivary complex studied in mouse brain slice.. J Neurosci.

[25] Haustein MD, Reinert T, Warnatsch A, Englitz B, Dietz B (2008). Synaptic transmission and short-term plasticity at the calyx of Held synapse revealed by multielectrode array recordings.. Journal of Neuroscience Methods.

[26] Hodgkin AL, Huxley AF (1952). A quantitative description of membrane current and its application to conduction and excitation in nerve.. J Physiol (Lond).

[27] Zhang X, Heinz MG, Bruce IC, Carney LH (2001). A phenomenological model for the responses of auditory-nerve fibers: I. Nonlinear tuning with compression and suppression.. J Acoust Soc Am.

[28] Joris PX, Carney LH, Smith PH, Yin TCT (1994). Enhancement of neural synchronization in the anteroventral cochlear nucleus. I. Responses to tones at the characteristic frequency.. J Neurophysiol.

[29] Paolini AG, FitzGerald JV, Burkitt AN, Clark GM (2001). Temporal processing from the auditory nerve to the medial nucleus of the trapezoid body in the rat.. Hear Res.

[30] Tollin DJ, Yin TCT (2005). Interaural phase and level difference sensitivity in low-frequency neurons in the lateral superior olive.. J Neurosci.

[31] Tsuchitani C (1997). Input from the medial nucleus of trapezoid body to an interaural level detector.. Hear Res.

[32] Kopp-Scheinpflug C, Tolnai S, Malmierca MS, Rübsamen R (2008). The medial nucleus of the trapezoid body: comparative physiology.. Neuroscience.

[33] Englitz B (2009). Synaptic Transmission and Signal Representation at the Calyx of Held. Doctoral Thesis (Univ. of Leipzig)..

[34] Moore JK, Moore RY (1987). Glutamic acid decarboxylase-like immunoreactivity in brainstem auditory nuclei of the rat.. J Comp Neurol.

[35] Saint Marie RL, Benson CG, Ostapoff EM, Morest DK (1991). Glycine immunoreactive projections from the dorsal to the anteroventral cochlear nucleus.. Hear Res.

[36] Kolston J, Osen KK, Hackney CM, Ottersen OP, Storm-Mathisen J (1992). An atlas of glycine- and GABA-like immunoreactivity and colocalization in the cochlear nuclear complex of the guinea pig.. Anat Embryol.

[37] Juiz JM, Helfert RH, Bonneau JM, Wenthold RJ, Altschuler RA (1996). Three classes of inhibitory amino acid terminals in the cochlear nucleus of the guinea pig.. J Comp Neurol.

[38] Mahendrasingam S, Wallam CA, Polwart A, Hackney CM (2004). An immunogold investigation of the distribution of GABA and glycine in nerve terminals on the somata of spherical bushy cells in the anteroventral cochlear nucleus of guinea pig.. Eur J Neurosci.

[39] Wu SH, Oertel D (1986). Inhibitory circuitry in the ventral cochlear nucleus is probably mediated by glycine.. J Neurosci.

[40] Wickesberg RE, Oertel D (1990). Delayed, frequency-specific inhibition in the cochlear nuclei of mice: a mechanism for monaural echo suppression.. J Neurosci.

[41] Caspary DM, Backoff PM, Finlayson PG, Palombi PS (1994). Inhibitory inputs modulate discharge rate within frequency receptive fields of anteroventral cochlear nucleus neurons.. J Neurophysiol.

[42] Ebert U, Ostwald J (1995). GABA can improve acoustic contrast in the rat ventral cochlear nucleus.. Exp Brain Res.

[43] Joris PX, Smith PH, Yin TC (1994). Enhancement of neural synchronization in the anteroventral cochlear nucleus. II. Responses in the tuning curve tail.. J Neurophysiol.

[44] Cant NB, Morest DK (1979). The bushy cells in the anteroventral cochlear nucleus of the cat. A study with the electron microscope.. Neuroscience.

[45] Rothman JS, Young ED, Manis PB (1993). Convergence of auditory nerve fibers onto bushy cells in the ventral cochlear nucleus: implications of a computational model.. J Neurophysiol.

[46] Rothman JS, Young ED (1996). Enhancement of Neural Synchronisation in Computational Models of Ventral Cochlear Nucleus Bushy Cells.. Auditory Neurosci.

[47] Sätzler K, Söhl LF, Bollmann JH, Borst JGG, Frotscher M (2002). Three-dimensional reconstruction of a calyx of Held and its postsynaptic principal neuron in the medial nucleus of the trapezoid body.. J Neurosci.

[48] Kuwabara N, DiCaprio RA, Zook JM (1991). Afferents to the medial nucleus of the trapezoid body and their collateral projections.. J Comp Neurol.

[49] Kuwabara N, Zook JM (1991). Classification of the principal cells of the medial nucleus of the trapezoid body.. J Comp Neurol.

[50] Smith PH, Joris PX, Carney LH, Yin TCT (1991). Projections of physiologically characterized globular bushy cell axons from the cochlear nucleus of the cat.. J Comp Neurol.

[51] Banks MI, Smith PH (1992). Intracellular recordings from neurobiotin-labeled cells in brain slices of the rat medial nucleus of the trapezoid body.. J Neurosci.

[52] Turecek R, Trussell LO (2001). Presynaptic glycine receptors enhance transmitter release at a mammalian central synapse.. Nature.

[53] Awatramani GB, Turecek R, Trussell LO (2004). Inhibitory control at a synaptic relay.. J Neurosci.

[54] Awatramani GB, Price GD, Trussell LO (2005). Modulation of transmitter release by presynaptic resting potential and background calcium levels.. Neuron.

